# Reply: “ *PGRN* Manifesting as Parkinson's Disease: Counseling and Treatment Implications”

**DOI:** 10.1002/mdc3.70326

**Published:** 2025-09-15

**Authors:** Angelo Antonini, Giulia Bonato, Miryam Carecchio

**Affiliations:** ^1^ Neurodegenerative Disease Unit, Centre for Rare Neurological Diseases (ERN‐RND), Department of Neuroscience, Padua Neuroscience Center (PNC) University of Padova Padova Italy; ^2^ IRCCS San Camillo Venice Venice Italy

**Keywords:** genetics, GRNparkinsonism

Thank you for your interest in our case series, “Progranulin Mutation Manifesting as Parkinson Disease: From the PADUA‐CESNE Cohort,” and for sharing this valuable additional case.^1^

We appreciate this detailed description, along with the illustrative video, of a patient with a progranulin (PGRN) mutation presenting with a tremor‐dominant Parkinson's disease phenotype and remarkably intact cognition 11 years after onset.^2^ This case indeed strengthens our suggestions and further highlights the phenotypic heterogeneity of PGRN mutation carriers. Mutations in the GRN gene lead to reduced PGRN levels, and this deficiency has been linked to increased α‐synuclein aggregation and worsened disease progression in tauopathy models. The influence of PGRN on synucleinopathies appears to be mediated through its interaction with lysosomal function, particularly the autophagy‐lysosome pathway and glucocerebrosidase activity.^3^

The observation regarding the absence of progression to frontotemporal dementia in this individual, even after significant disease duration, strongly supports the need for adequate genetic counseling for PGRN mutation carriers addressing also the possibility of developing Parkinson's symptoms. This also applies to other mutations and variants that may be considered Parkinson's mimics, which are detected with varying frequency across many cohort studies, including ours.^4^ We believe this is especially important because current research is intensively targeting α‐synuclein aggregation, whereas in the rarer genetic variants of the disorder, it may be more effective to tackle discrete molecular pathways linked to the degenerative process.^5^

## Disclosure

**Ethical Compliance Statement:** The authors confirm that the approval of an institutional review board was not required for this work. Informed patient consent was not necessary for this work. We confirm that we have read the Journal's position on issues involved in ethical publication and affirm that this work is consistent with those guidelines.

**Funding Sources and Conflict of Interest:** No specific funding was received for this work. The authors declare that there are no conflicts of interest relevant to this work.

**Financial Disclosures for the Previous 12 Months:** A.A. has received compensation for consultancy and speaker‐related activities from AbbVie, Britannia, Zambon, Bial, Theravance Biopharma, Ever Pharma, Bayer, Ferrer, and AskBio. He receives research support from Horizon2020, Ministry of University and Research, Ministry of Health, European Union ‐ NextGenerationEU–NRRP M6C2 ‐ Investment: 2.1 “Enhancement and strengthening of biomedical research within the NSH.” G.B. and M.C. declare no conflicts of interest.

## References

[1] Bonato G , Campagnolo M , Emmi A , et al. Progranulin mutation manifesting as Parkinson disease: a case series from the PADUA‐CESNE cohort. Mov Disord Clin Pract 2025;12:998–1002. 10.1002/mdc3.70064. 40183420 PMC12275000

[2] Stamelou M . PGRN manifesting as Parkinson's disease: counselling and treatment implications. Mov Disord Clin Pract 2026;13(2):583–584.10.1002/mdc3.7032840948304

[3] Fujimori H , Ohba T , Nakamura S , Shimazawa M , Hara H . The involvement of progranulin for α‐synuclein reduction through autolysosome formation. Biol Pharm Bull 2023;46(8):1032–1040. 10.1248/bpb.b22-00711. 37532554

[4] Bonato G , Antonini A , Pistonesi F , et al. Genetic mutations in Parkinson's disease: screening of a selected population from North‐Eastern Italy. Neurol Sci 2025;46(1):165–174. 10.1007/s10072-024-07690-7. 39034353 PMC11698772

[5] Stocchi F , Bravi D , Emmi A , Antonini A . Parkinson disease therapy: current strategies and future research priorities. Nat Rev Neurol 2024;20(12):695–707. 10.1038/s41582-024-01034-x. 39496848

